# Comparative Evaluation of Nephrotoxicity and Management by Macrophages of Intravenous Pharmaceutical Iron Formulations

**DOI:** 10.1371/journal.pone.0125272

**Published:** 2015-05-14

**Authors:** James R. Connor, Xuesheng Zhang, Anne M. Nixon, Becky Webb, Joseph R. Perno

**Affiliations:** 1 Department of Neurosurgery, M.S. Hershey Penn State University College of Medicine, M.S. Hershey Medical Center, Hershey, Pennsylvania, United States of America; 2 Luitpold Pharmaceuticals, Inc., Norristown, Pennsylvania, United States of America; Lady Davis Institute for Medical Research/McGill University, CANADA

## Abstract

**Background:**

There is a significant clinical need for effective treatment of iron deficiency. A number of compounds that can be administered intravenously have been developed. This study examines how the compounds are handled by macrophages and their relative potential to provoke oxidative stress.

**Methods:**

Human kidney (HK-2) cells, rat peritoneal macrophages and renal cortical homogenates were exposed to pharmaceutical iron preparations. Analyses were performed for indices of oxidative stress and cell integrity. In addition, in macrophages, iron uptake and release and cytokine secretion was monitored.

**Results:**

HK-2 cell viability was decreased by iron isomaltoside and ferumoxytol and all compounds induced lipid peroxidation. In the renal cortical homogenates, lipid peroxidation occurred at lowest concentrations with ferric carboxymaltose, iron dextran, iron sucrose and sodium ferric gluconate. In the macrophages, iron sucrose caused loss of cell viability. Iron uptake was highest for ferumoxytol and iron isomaltoside and lowest for iron sucrose and sodium ferric gluconate. Iron was released as secretion of ferritin or as ferrous iron via ferroportin. The latter was blocked by hepcidin. Exposure to ferric carboxymaltose and iron dextran resulted in release of tumor necrosis factor α.

**Conclusions:**

Exposure to iron compounds increased cell stress but was tissue and dose dependent. There was a clear difference in the handling of iron from the different compounds by macrophages that suggests in vivo responses may differ.

## Introduction

Severe iron deficiency is a common complication of gastric bypass surgery, chemotherapy, radiotherapy and chronic kidney disease. In addition, conditions such as heavy uterine bleeding and postpartum anemia can be associated with significant iron deficiency anemia that requires clinical intervention. Currently, the effective treatment options for iron deficiency anemia, especially among those individuals with a significant disease burden, are limited. Oral iron compounds tend to be ineffective and associated with gastrointestinal side effects that limit compliance. There has been considerable effort in the development of intravenous iron therapy, and some success in treating iron deficiency anemia has been achieved [1,2]. Concerns remain, however, about the safety associated with the relatively large doses of iron that must be given to achieve reversal of iron deficiency [3]. Iron, although essential for normal metabolic functions and energy production, is a transition element and this property makes it capable of generating oxygen free radicals [4, 5, 6], the precursor of oxidative damage. The relative concentration of iron in the body makes this metal the most potent inducer of oxidative stress in biological systems [7,8]. Thus, concerns over the delivery of high concentrations of iron require investigation.

The mechanism by which the body handles intravenous iron injections is thought to begin in the reticuloendothelial system [9]. Macrophages are known to acquire iron under multiple physiological and clinical conditions through phagocytosis [10,11], but the release of that iron to participate in iron replenishment is poorly if at all understood. Therefore we examined the ability of the macrophages to acquire iron from various iron formulations that are in use clinically. The release of iron by the macrophages as an indication of bioavailability of the iron was also determined. Moreover, because iron accumulation in macrophages is a common occurrence in disease states [12,13] and influences their inflammatory status [14] we determined the impact of the different iron formulations on cytokine release by the macrophages.

A significant target of the intravenously delivered iron compounds is the kidney. A vast majority of metabolic products as well as many pharmacological compounds are excreted via the kidney. The kidney is a lipid rich organ and therefore potentially more vulnerable to iron induced oxidative damage. Within the kidney, the proximal tubule is one of the main sites of nephrotoxicity. Therefore, in this study, we utilized primary rat renal cortical homogenates and a human kidney cell line (HK-2) to evaluate the potential for induction of oxidative stress among the pharmaceutical iron formulations.

## Materials and Methods

### Chemicals

Chemicals and materials were obtained from the following sources: Keratinocyte-serum free medium, Trypan blue, Trypsin/EDTA, and antibiotic/antimycotic were obtained from Gibco BRL (Grand Island, NY), fetal bovine serum (FBS) was purchased from Biocell (Rancho Dominguez, CA), calcium- and magnesium-free Hank’s buffered salt solution (CMF-HBSS) were obtained from Life Technology (Gaithersburg, MD), and all other chemicals were obtained from Sigma (St. Louis, MO).

### Iron Preparations

The compounds used in this study were DexFerrum: iron dextran (American Regent, INC, Shirley, NY), Ferrlecit: sodium ferric gluconate complex in sucrose (Watson Pharma, Inc. Corona, CA), Venofer: iron sucrose (American Regent Laboratories, Inc., Shirley, NY), ferric carboxymaltose (American Regent Laboratories, Inc., Shirley NY); Monofer: iron isomaltoside (Pharmacosmos), and Ferumoxytol (AMAG Pharmaceuticals). The concentrations (administered doses) ranged from 0.1 to 0.8 mg/ml were determined empirically to be sublethal in cell culture and were also chosen to fall within clinically relevant human doses [15].

### Human Kidney Cell Line

The HK-2 cell line, an immortalized proximal tubule epithelial cell line from normal adult human kidney, was purchased from American Type Culture Collection (ATCC). Cells were maintained in Keratinocyte-serum free medium with 10% fetal bovine serum (FBS), 1% streptomycin and penicillin, 5 ng/ml recombinant epidermal growth factor, 0.05 mg/ml bovine pituitary extract, and incubated at 37°C under an atmosphere of 5% CO2 with humidified air. The HK-2 cells were grown to 85–95% confluency and then washed with Hanks balanced salt solution, trypsinized with 5 ml of 0.25% (w/v) EDTA, diluted, counted, and seeded in 24-well tissue culture plates (5 x 10^5^ cells/well). After 48 hours of incubation, the medium growth medium was removed and replaced with Keratinocyte-serum free medium.

### Cell Viability Experiments

Following the replacement of serum free media, the HK-2 cultures equilibrated for 24 hours in this medium and then were exposed to the different iron formulations in doses ranging from 0 to 0.8 mg/ml for 24 hours. Cell viability was determined by the MTT assay and plasma membrane integrity evaluated by assaying for LDH in the media according to manufacturer instructions.

### Oxidative Stress Assay

LPO (lipid peroxidation) content of the HK-2 cells exposed to the different iron formulations, in doses ranging from 0 to 0.8 mg/ml for 24 hours, was measured by a colorimetric microplate assay kit (Oxford Biomedical Research, Product No. FR22). The extent of protein oxidation was determined by the carbonyl protein content using the Oxiselect Protein Carbonyl ELISA kit, with a detection limit of 10 μg/ml (Cell Biolabs, INC. catalog number STA-310-5).

### Rat Peritoneal Macrophage Cultures

Rat peritoneal macrophages were obtained by peritoneal lavage and then washed twice with DMEM, resuspended at a concentration of 10^6^ cells/ml in DMEM supplemented with 10% fetal bovine serum, 300 μg/ml glutamine, 100 U/ml penicillin and 100 μg/ml streptomycin and plated in six-well dishes. Forty-eight hours later, nonadherent cells were removed by washing with fresh medium, and the remaining cells comprised primarily of macrophages, as determined using immunostaining for OX-42 and Ibal (Wako) [16,17], were used for the experiments. For the iron exposure experiments, the cells were washed in DMEM and then maintained in DMEM with 1% fetal bovine serum for 24 hours prior to iron compound exposure and throughout the remainder of the experiment.

### Immunoblot Analysis for Ferroportin and H-ferritin

For these studies, 0.1 mg/ml of elemental iron from the different iron formulations was added to the media of the peritoneal macrophages. After 24 hours, the cells were washed with cold PBS and lysed in lysis buffer (50 mM Tris—HCl, pH 8.0, 150 mM NaCl, 0.02% sodium azide, 1% Nonidet P-40, 1 mg/ml aprotinin, 1 mM phenylmethylsulfonyl fluoride, 1 mM sodium metavanadate, 5 mM sodium fluoride) containing protease inhibitors (NaVO3). The protein concentration in cell lysates was determined using a BCA protein assay reagent kit (Pierce, Rockford, IL). A total of 20 μg of protein was loaded onto a 4–20% gradient gel and electrophoresis applied for 2 hours at 120V using Tris-Glycine buffer. The protein was transferred to nitrocellulose membrane using standard procedures. The nitrocellulose membrane was blocked in 5% blotto (in TBST, pH7.5) for 30 minutes followed by incubation with H-ferritin antibody (Mouse polyclonal from Abcam 1:1000) or ferroportin antibody (Rabbit polyclonal from Abcam 1:1000) for 16 hours. The membranes were rinsed 3x for 15 min each in TBST, and then incubated in anti-mouse or anti-rabbit secondary antibody conjugated with HRP (1:5000). The membrane was exposed to ECL reagent for 5 minutes and images collected on a FUJI system (10 minute exposure). In addition, the expression levels were normalized to β-actin.

### H-ferritin Secretion and Iron Release

After 0.1 mg/ml of elemental iron from the different iron compounds was added to the medium for 24 hours, the medium was changed to 1% fetal bovine serum for another 24 hours. The media was then collected and concentrated ten-fold by centrifuging samples in Centricon Ultracel YM-10 centrifugal filter devices (Millipore, Billerica, MA, USA) at 5,000 ×g (~20 min, 4°C), and frozen in small aliquots held at −80°C.

Total iron and ferrous iron content in media was also determined after exposure to the iron formulations with or without hepcidin (Peptides International Inc. PLP-4392-s) treatment. The iron concentration in the media was measured using the QuantiChrom iron assay kit (DIFE-250).

### Labile Iron Pool Measurement

Labile iron pool (LIP) of the rat peritoneal macrophages was measured using a Calcein AM Assay (Trevigen, Gaithersbug, MD). The macrophages (5000 cells/well) were added to a black 96-well cell culture plate for 48 hours and then 0.1 mg/ml elemental iron from the different iron compounds was added to each of the wells for 24 hours. The media was then removed and cells were washed with 1X Calcein AM wash buffer and incubated with 50 μl of 1 μM Calcein AM working solution and 50 μl 1X Calcein AM wash buffer for 30 minutes at 37°C under 5% CO2. Fluorescence was quantified on a Gemini EM fluorescent plate reader (Molecular Devices) at excitation/emission wavelengths of 490/520 nm.

### Oxidative Stress Analysis within Mitochondria and Cytoplasm

Oxidative stress within the rat peritoneal macrophage cytoplasm and mitochondria was measured using ROS sensitive dyes, CellROX Deep Red and Green Reagents, respectively (Life Technologies, Carlsbad, CA). The macrophages (5000 cells/well) were added to a black 96-well cell culture plate for 48 hours and then 0.1 mg/ml elemental iron from the different iron compounds was added to each of the wells for 24 hours. Subsequently, either CellROX Deep Red Reagent or CellROX Green Reagent was added directly to the media while the iron compounds were still present and incubated for 30 minutes at 37°C under 5% CO2. The media was then removed and cells were washed with three times with 1X PBS. Fluorescence was quantified on a Gemini EM fluorescent plate reader (Molecular Devices) at excitation/emission wavelengths of 640/665 nm for CellROX Deep Red and 485/520 nm for CellROX Green.

### Cytokine Release

The same media as used for ferritin and iron concentration was used to determine cytokine expression. For the cytokine assays, an aliquot of the media was collected, filtered with a 0.45 μm filter, aliquoted, and stored at -20°C until use. All medium was used within a week. IL-1β, TNF-α, and IL-6 protein was examined by an ELISA kit.

### Renal Cortical Homogenates

These studies were approved by the IACUC at Penn State Hershey Medical Center. Animals were killed by decapitation. The kidneys were then quickly removed into a solution of cold CMF-HBSS (4°C) and then washed using cold CMF-HBSS. The connective tissue was removed in CMF-HBSS and the renal cortex dissected free from the rest of kidney. The renal cortex was weighed and homogenized on ice within 10 minutes of dissection. The homogenate was diluted to 100 mg/ml. The homogenates were centrifuged at 4000 ×g at 4°C for 10 minutes to yield a low speed supernatant fraction that was used immediately for assay or kept it in -70°C.

### Oxidative Stress Assay

Lipid peroxidation content of the cells was measured by a colorimetric microplate assay kit (Oxford Biomedical Research, Product No. FR22), following exposure to the different iron formulations. The extent of protein oxidation was determined by the carbonyl protein content using the Oxiselect Protein Carbonyl ELISA kit, with a detection limit of 10 μg/ml (Cell Biolabs, INC. catalog number STA-310-5).

### Calculations and Statistics

All values are presented as mean +/-SEM. Statistical comparisons among the values obtained for each group were made by grouped t-test or one-way analysis of variance (ANOVA) followed by Dunnett’s test for multiple group comparisons with a single control group when applicable. A p-value of <0.05 was considered statistically significant.

## Results

### Human Kidney Cell Line

**Fig 1:**
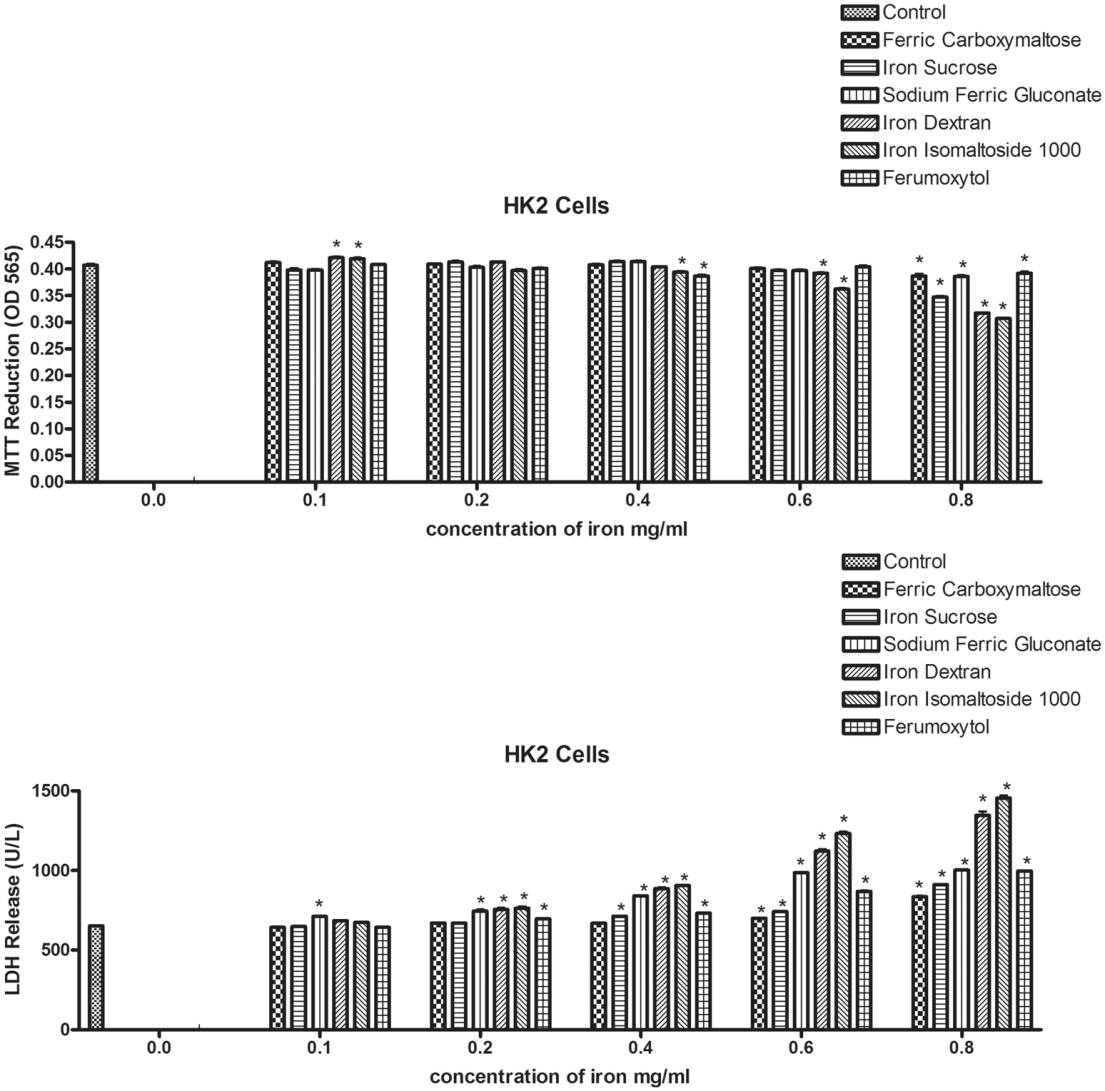
Cytotoxicity profile of six iron compounds on HK-2 cells. The cells were exposed to the different iron compounds in doses ranging from 0.1 to 0.8 mg/ml for 24 hours. During the final 4 hours, MTT was added (top figure, A). The MTT assay was carried out to establish cell viability. In a separate set of experiments from those used for the MTT assays, cells were exposed to the different iron compounds at the ranges indicated for 24 hours. In a separate set of experiments, plasma membrane integrity was evaluated by LDH assay. The amount of lactate dehydrogenase (LDH) released from the cells was compared to the control group (bottom figure, B).. Data represents the mean±SEM from five independent experiments. *: compared to the control p<0.05.

To determine if the iron formulations were toxic to cells, the HK-2 cell line was exposed to 5 different concentrations of the various iron compounds for 24 hours. Cytotoxicity was determined using both the MTT and LDH assays. In the MTT assay, iron dextran and iron isomaltoside showed increased cell viability compared to the control at the lowest concentration. Significant toxicity was not observed until the 0.4 mg/ml and was limited to iron isomaltoside and ferumoxytol. At the highest concentration, all of the compounds were associated with cellular toxicity (Fig 1A).

LDH is a cytosolic protein that is released into the medium when the plasma membrane is compromised. When the HK-2 cells were exposed to the different iron formulations, significant increased levels of LDH were observed in the medium for sodium ferric gluconate at the lowest concentration. Within a clinically relevant dosing range all of the compounds, except ferric carboxymaltose were associated with significant release of LDH. Ferric carboxymaltose was not associated with LDH release until the cells were exposed to relatively higher concentrations (Fig 1B).

**Fig 2:**
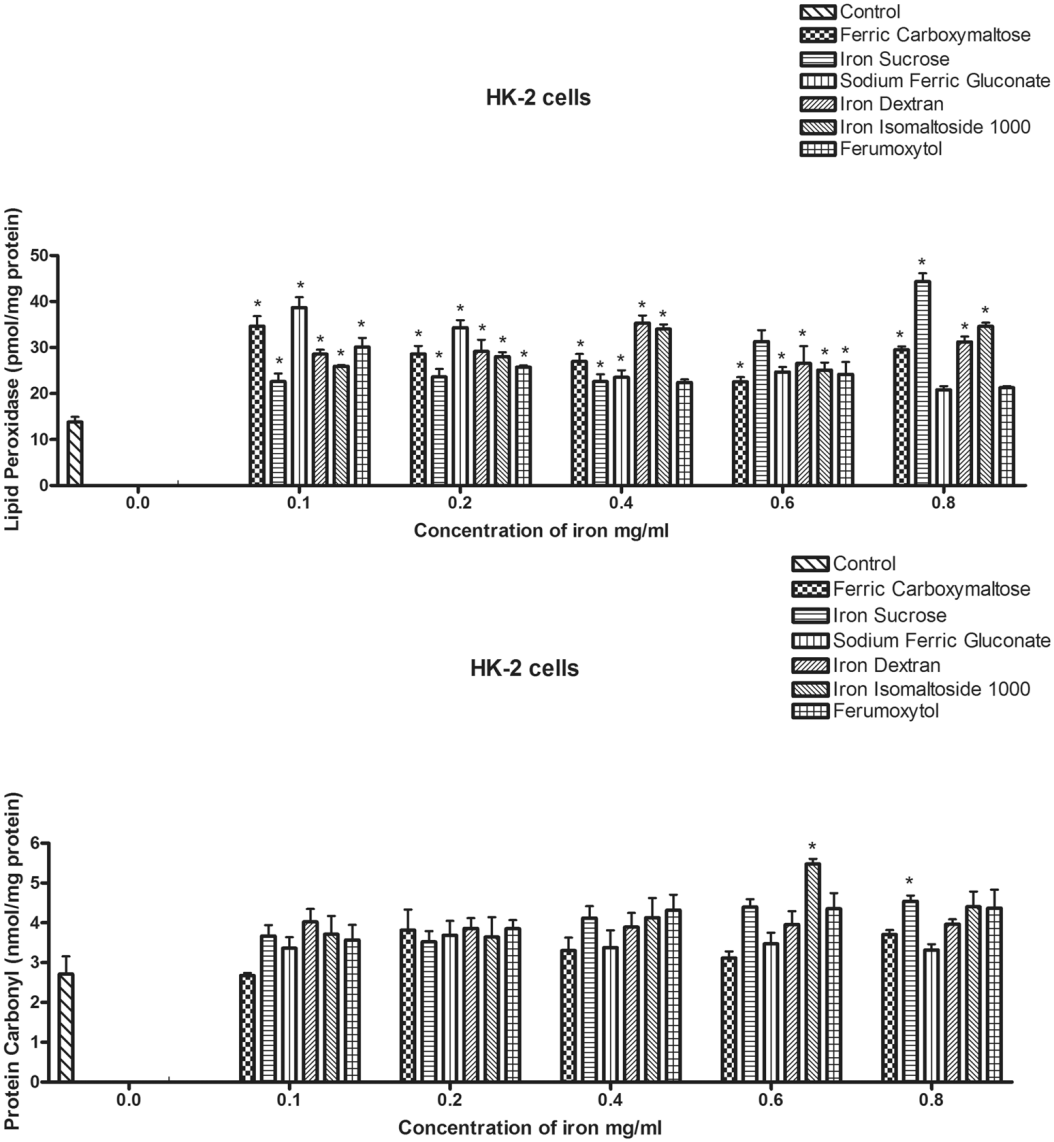
Effects of the iron compounds on markers of oxidative stress in HK-2 cells. HK-2 cells were seeded in T-75 culture flask (5 x 10^6^ cells/flask). After 48 h of incubation, the medium was removed and replaced with Keratinocyte-serum free medium. The cells were exposed to the different iron compounds in doses ranging from 0.1 to 0.8 mg/ml for 24 hours. After 24 hours, cells were harvested and lysed for lipid peroxidation analysis (top figure, A) or assayed for oxidative modification to the proteins using the carbonyl assay (bottom figure, B). Data represents the mean±SEM from three independent experiments and are compared to the control for statistical significance using 2- way ANOVA and post-hoc testing. *p<0.05.

To evaluate the ability of the compounds to induce oxidative stress, we evaluated lipid peroxidation (LPO, Fig 2A) and protein oxidation (carbonyl levels, Fig 2B). The level of LPO was significantly increased beginning at the lowest concentration examined (0.1 mg/ml) for all of the iron formulations (Fig 2A). Evidence of protein oxidation, as determined by presence of carbonyl groups, was not detected following exposure to any of the formulations until higher concentrations (0.6 mg/ml) of iron isomaltoside and iron sucrose (0.8 mg/ml) (Fig 2B).

### Renal Cortical Homogenates

**Fig 3:**
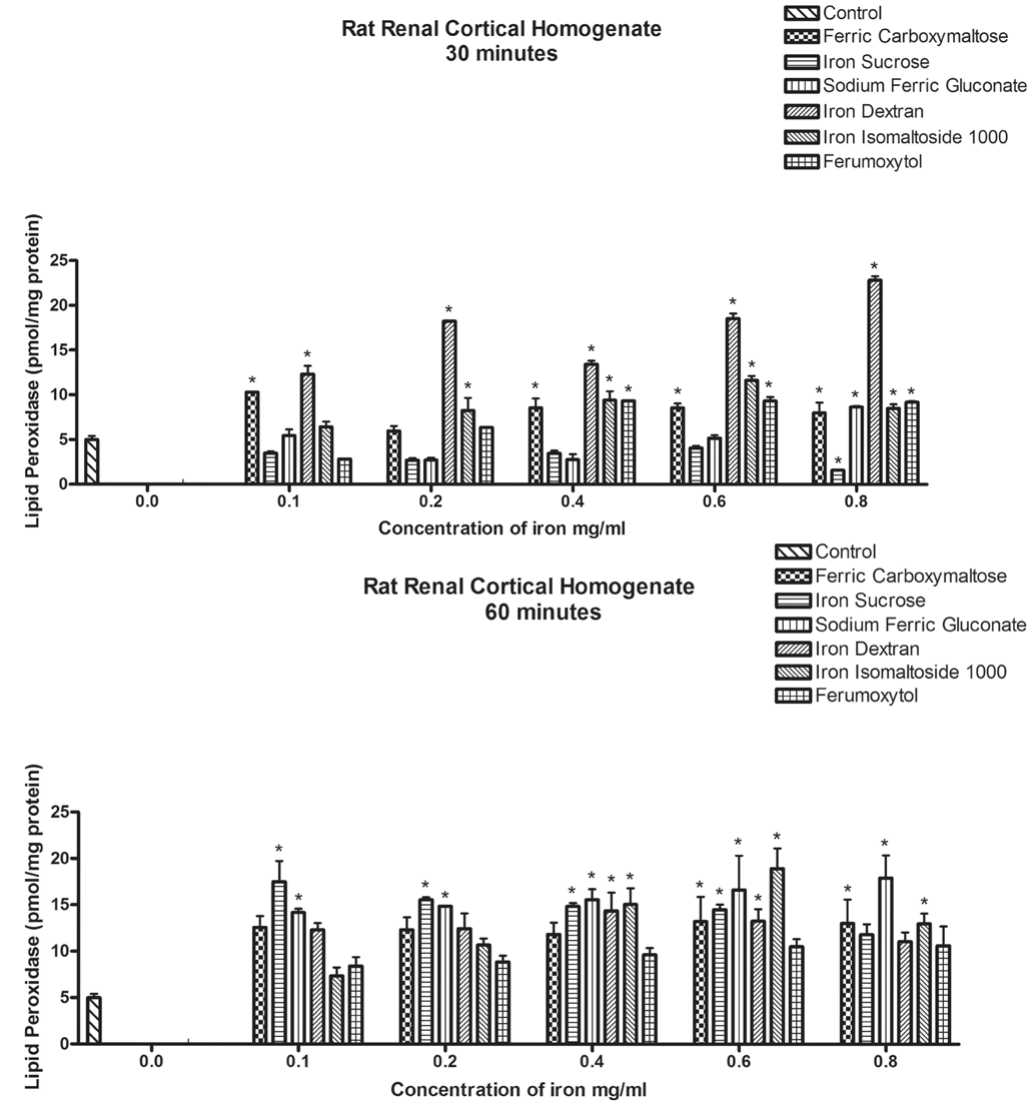
Induction of lipid peroxidation by iron formulations on rat renal cortical homogenates. The top figure (A) shows the effect of exposing the homogenates to the different iron formulation for 30 minutes. The result of exposure for 60 minutes is shown in the bottom figure (B). Results are the mean of three separate experiments, expressed as micromoles of LPO per milligram of renal cortical tissue. Data represents the mean±SEM from three independent experiments and are compared to control for statistical significance using 2-way ANOVA and post-hoc testing. *p<0.05.

To assess the effect of exposure of iron compounds on primary tissue, rat renal cortical homogenates were used. The assessments were restricted to lipid and protein oxidative stress and two time periods were examined, 30 and 60 minutes. Lipid peroxidative damage was evident in the ferric carboxymaltose and iron dextran exposed groups at the lowest concentration (0.1 mg/ml) within 30 minutes (Fig 3A). The degree of lipid peroxidation was greatest for iron dextran at each concentration. Lipid peroxidation was first seen for iron isomaltoside at 0.2 mg/ml. At 0.4–0.6 mg/ml, only iron sucrose and sodium ferric gluconate exposure was not associated with lipid peroxidation, until the highest iron concentration (Fig 3A).

At 60 minutes of exposure, iron sucrose and sodium ferric gluconate were associated with increased lipid peroxidation beginning with the lowest concentration and extending through all doses tested. At the 0.4 mg/ml concentration, iron dextran and iron isomaltoside also caused lipid peroxidation. Ferric carboxymaltose exposure was not associated with lipid peroxidative damage until 0.6 mg/ml concentration (Fig 3B).

**Fig 4:**
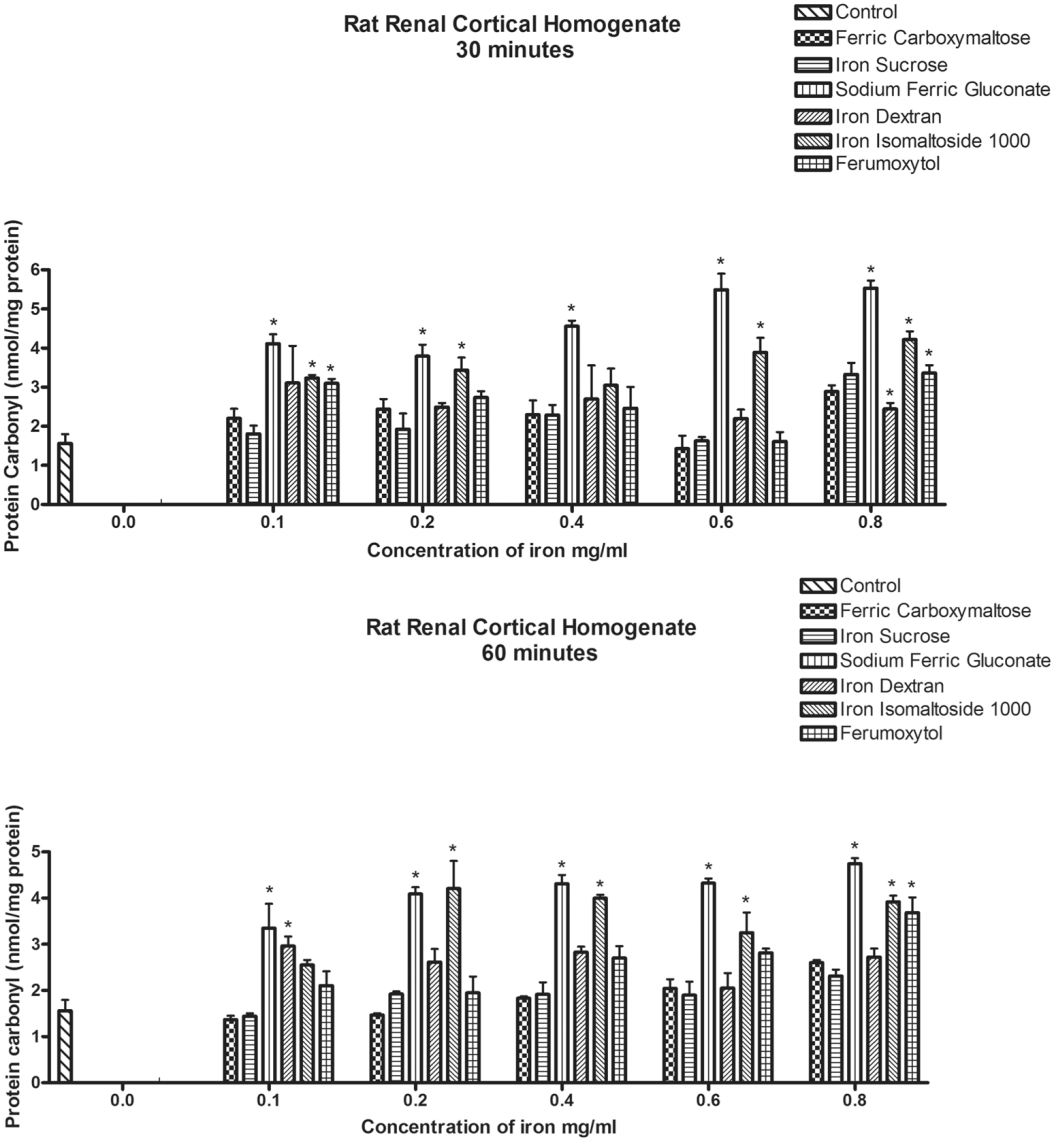
Effects of exposure of the iron compounds to Rat Renal Cortical Homogenates on expression of oxidatively modified proteins. The top figure (A) shows the results for 30 minutes of exposure and in the bottom figure (B), the results for 60 minutes of exposure. Data represents the mean±SEM from three independent experiments and are compared to control for statistical significance using ANOVA and post-hoc testing. *p<0.05.

Oxidative modification to proteins was examined in the same tissue samples by carbonyl assay. Following 30 minutes of exposure at the lowest concentration of iron compound (0.1 mg/ml), sodium ferric gluconate, iron isomaltoside and ferumoxytol were associated with protein oxidative damage. At the highest concentration, iron dextran was also associated with protein oxidation. There was no evidence of protein oxidation at 30 minutes for either ferric carboxymaltose or iron sucrose at this time period at any concentration (Fig 4A).

In the homogenates exposed for 60 minutes, ferric carboxymaltose and iron sucrose were the only two formulations that did not increase protein oxidative damage at any concentration. Protein damage with ferumoxytol was only seen at the highest concentration and only at the lowest concentration for iron dextran (Fig 4B). Overall the pattern of protein oxidative damage and iron compounds was similar to that seen for 30 minutes of exposure (Fig 4A).

### Peritoneal Macrophages

**Fig 5:**
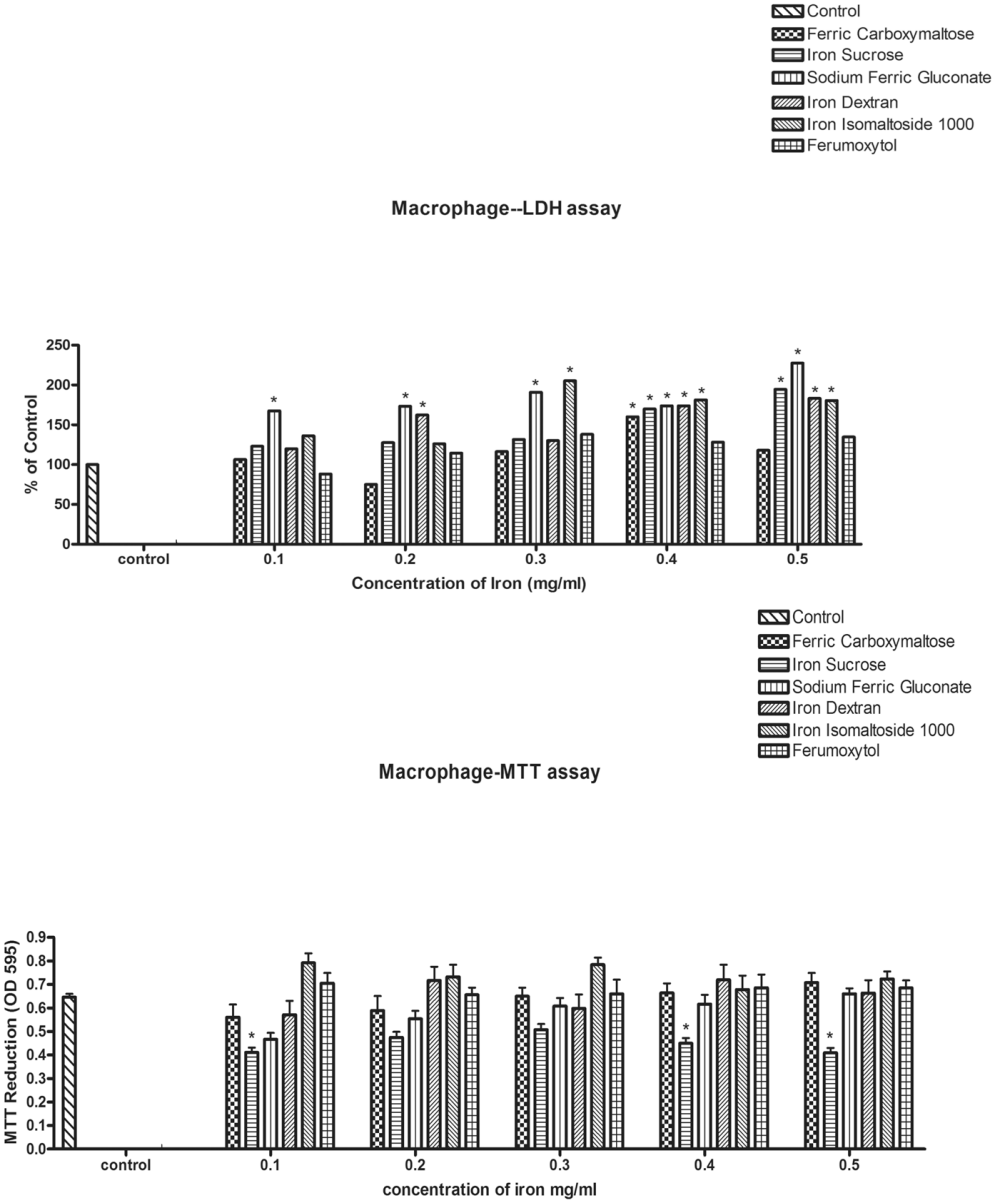
The cytotoxic effect of the iron compounds on rat primary peritoneal macrophages. Rat peritoneal macrophages were obtained by peritoneal lavage. The cells were exposed to the different concentrations of iron compounds for 24 hours. Lactate dehydrogenase activity in media and in cell lysates was measured (top figure, A). In a separate experiment the MTT substrate was added to the media during the last four hours (bottom figure, B). Results are means±SEM of three experiments, each conducted in triplicate. Data represents the mean±SEM from three independent experiments and are compared to control for statistical significance using ANOVA and post-hoc testing. *p<0.05.

The cytotoxicity of the iron formulations to peritoneal macrophages was assessed by LDH (Fig 5A) and MTT (Fig 5B) assays. Sodium ferric gluconate induced significant LDH release compared to control beginning at the lowest experimental dose and throughout all doses evaluated. Iron dextran increased LDH release beginning at 0.2 mg/ml and iron isomaltoside at 0.3 mg/ml. Iron sucrose became cytotoxic at 0.4 mg/ml.

In the MTT assay, only iron sucrose was associated with cytotoxicity. Iron isomaltoside and ferumoxytol had mild, but not statistically significant stimulatory effects at low concentrations (Fig 5B).

**Fig 6:**
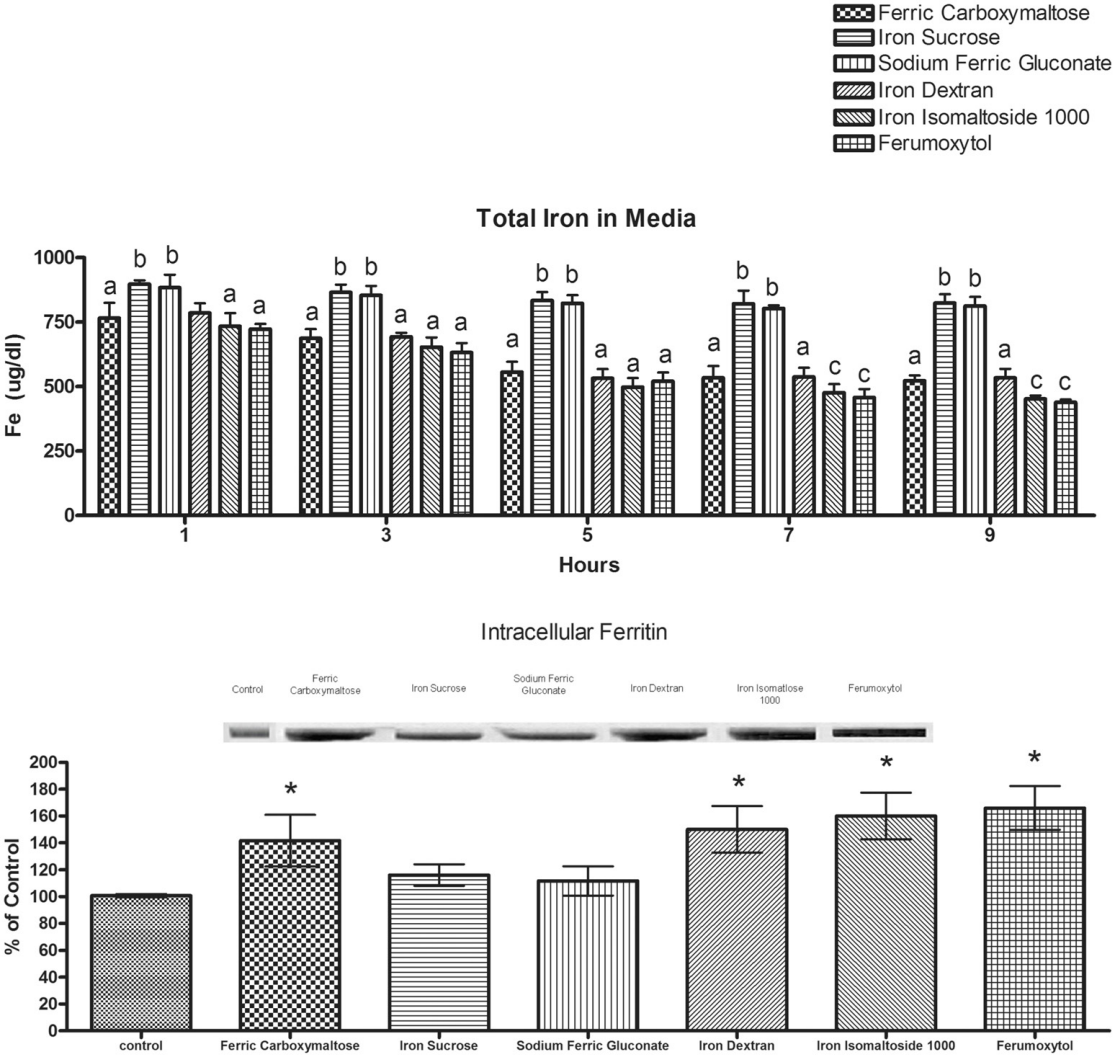
Total iron content in media and the content of H-ferritin in primary peritoneal macrophages after exposure to the iron formulations. Rat peritoneal macrophages were obtained by peritoneal lavage. The cells were exposed to 0.1 mg/ml of elemental iron for each of the iron formulations for 24 hours. The media was then monitored for presence of total iron by removing 50uL aliquots from the media over 9 hours. The top figure (A) shows total iron in the media was highest (indicating decreased uptake) for iron sucrose and sodium ferric gluconate (b = not statistically significantly different) at all time points. The other compounds were similar (a) until 7 hours when iron uptake was assessed greater for iron isomaltoside and ferumoxytol (c). There is almost no loss of iron from the media over the time monitored for iron gluconate or iron sucrose. At the end of the experiment, t macrophages were lysed and the amount of H-ferritin present in the cytosol was determined (bottom figure, B). A representative immunoblot for H-ferritin is shown. The bands appear at 21 kDa. The data in the graphs are the mean±SEM of three separate experiments. The asterisk (*) indicates a difference from the control group at p<0.05.

To compare the differences in uptake of the various iron formulations by the macrophages, total iron content in the media was determined over time. Iron uptake by macrophages was poorest for iron sucrose and sodium ferric gluconate. The iron uptake was similar for the other compounds for the first 5 hours of exposure. By 7–9 hours of exposure, iron isomaltoside and ferumoxytol had the highest amount of iron uptake from the media (Fig 6A). The uptake data are supported by the immunoblot analysis that shows an increase in intracellular ferritin expression by macrophages following exposure to ferric carboxymaltose, iron isomaltoside, ferrumoxytol and iron dextran compared to iron sucrose and sodium ferric gluconate. The latter two did not differ from control (Fig 6B). The increase in intracellular ferritin not only reports on the increased iron uptake but also would provide a mechanism for storing the iron inside the cell in a manner that would minimize its ability to engage in oxidative stress activity [18].

**Fig 7:**
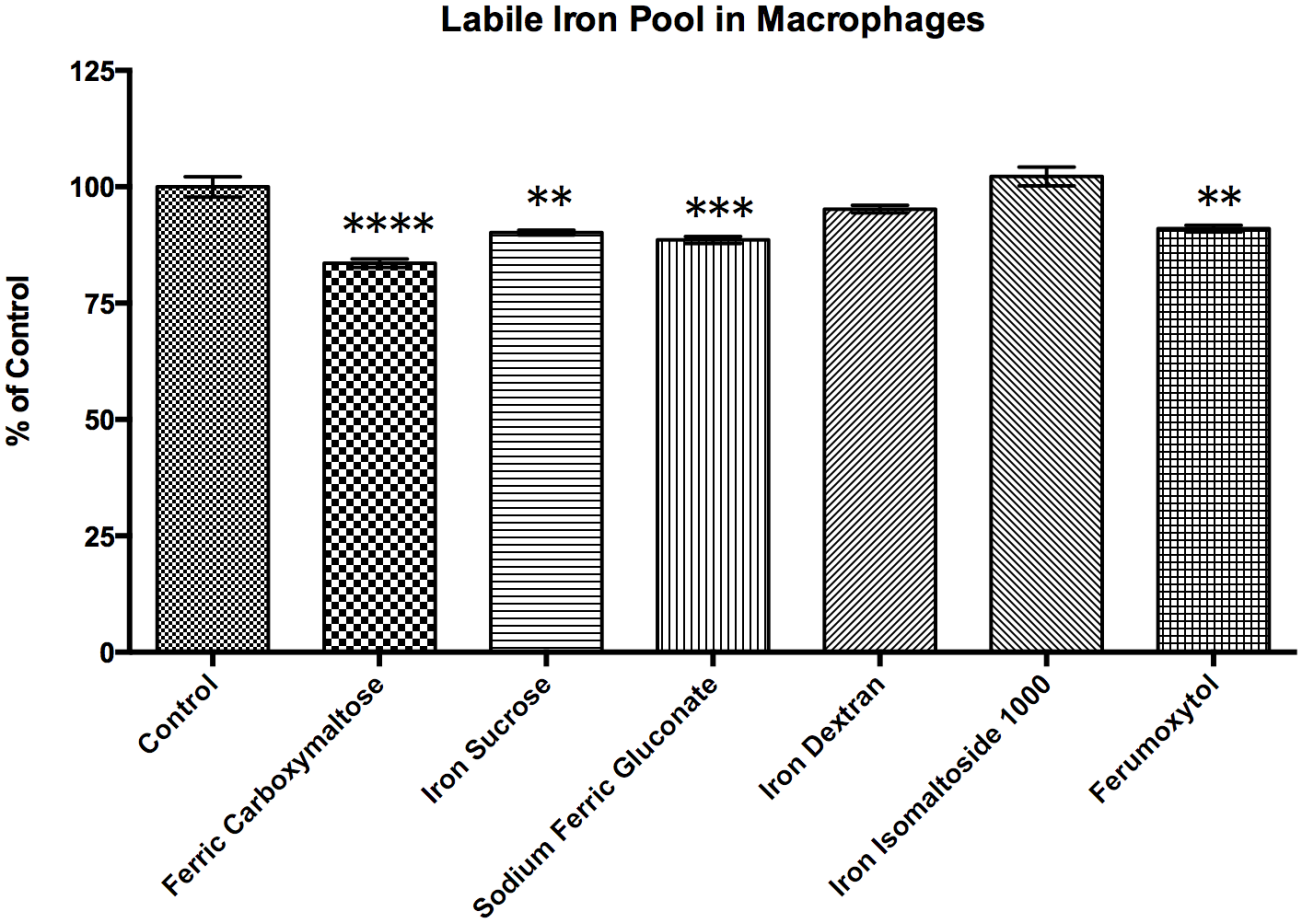
Labile Iron Pool assessment of rat peritoneal macrophages through Calcein AM Assay. Rat peritoneal macrophages were obtained by peritoneal lavage. The cells were exposed to 0.1 mg/ml of elemental iron for each of the iron formulations for 24 hours. After 24 hours, the cells were washed and incubated with Calcein AM working solution. Fluorescence was quantified on a fluorescent plate reader at excitation/emission wavelengths of 490/520 nm. When calcein binds to iron the fluorescent activity is quenched, thus more available iron is reported as decreased fluorescent activity. All data shown are means ± SEM of three independent observations in separate cell culture wells. The data show that exposure to ferric carboxymaltose, iron sucrose, sodium ferric gluconate, and ferumoxytol are associated with an increase in labile iron pool compared to control (untreated), but there is no significant increase following iron dextran or iron isomaltoside 1000 exposure (**, p<0.01; ***, p<0.001; ****, p<0.0001).

The uptake of iron was indirectly interrogated using calcein as a reporter for the labile iron pool. When calcein binds to iron the fluorescent activity is quenched, thus more available iron is reported as decreased fluorescent activity. Exposure to all of the iron compounds, except iron dextran and iron isomaltoside resulted in a significant increase in labile iron pool compared to the control (Fig 7).

**Fig 8:**
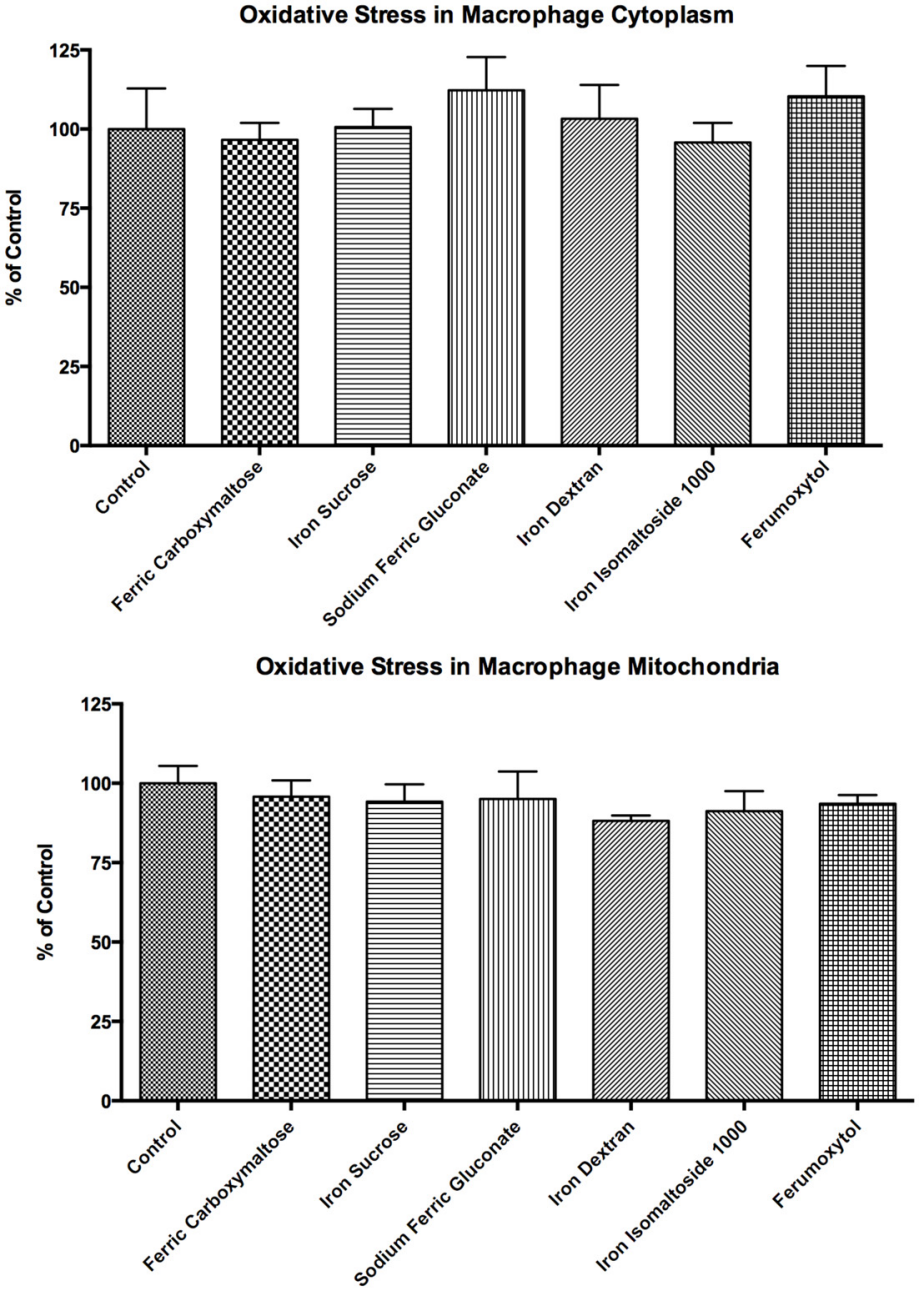
Oxidative stress within cytoplasm and mitochondria of rat macrophages. Rat peritoneal macrophages were obtained by peritoneal lavage. The cells were exposed to 0.1 mg/ml of elemental iron for each of the iron formulations for 24 hours. After 24 hours, the cells with iron were incubated with CellROX Deep Red Reagent (top figure, A) or CellROX Green Reagent (bottom figure, B) for thirty minutes. Following incubation, the cells were washed with PBS. Fluorescence was quantified on a fluorescent plate reader at excitation/emission wavelengths of 640/665 nm for cytoplasm study or 485/520 nm for mitochondria. All data shown are means ± SEM of three independent observations in separate cell culture wells. The data shows there were no significant changes in oxidative stress following the addition of the iron compounds compared to the control (untreated).

To determine if the addition of iron compounds resulted in oxidative stress, oxidative stress in the cytoplasm and mitochondria of rat peritoneal macrophages was evaluated using reactive oxygen species (ROS) dye (Fig 8A and 8B). The results indicate there were no significant changes in oxidative stress following the addition of iron compounds, supporting the proposed mechanism of increased ferritin minimizes oxidative stress activity.

### Release of Ferrous Iron into the Media

**Fig 9:**
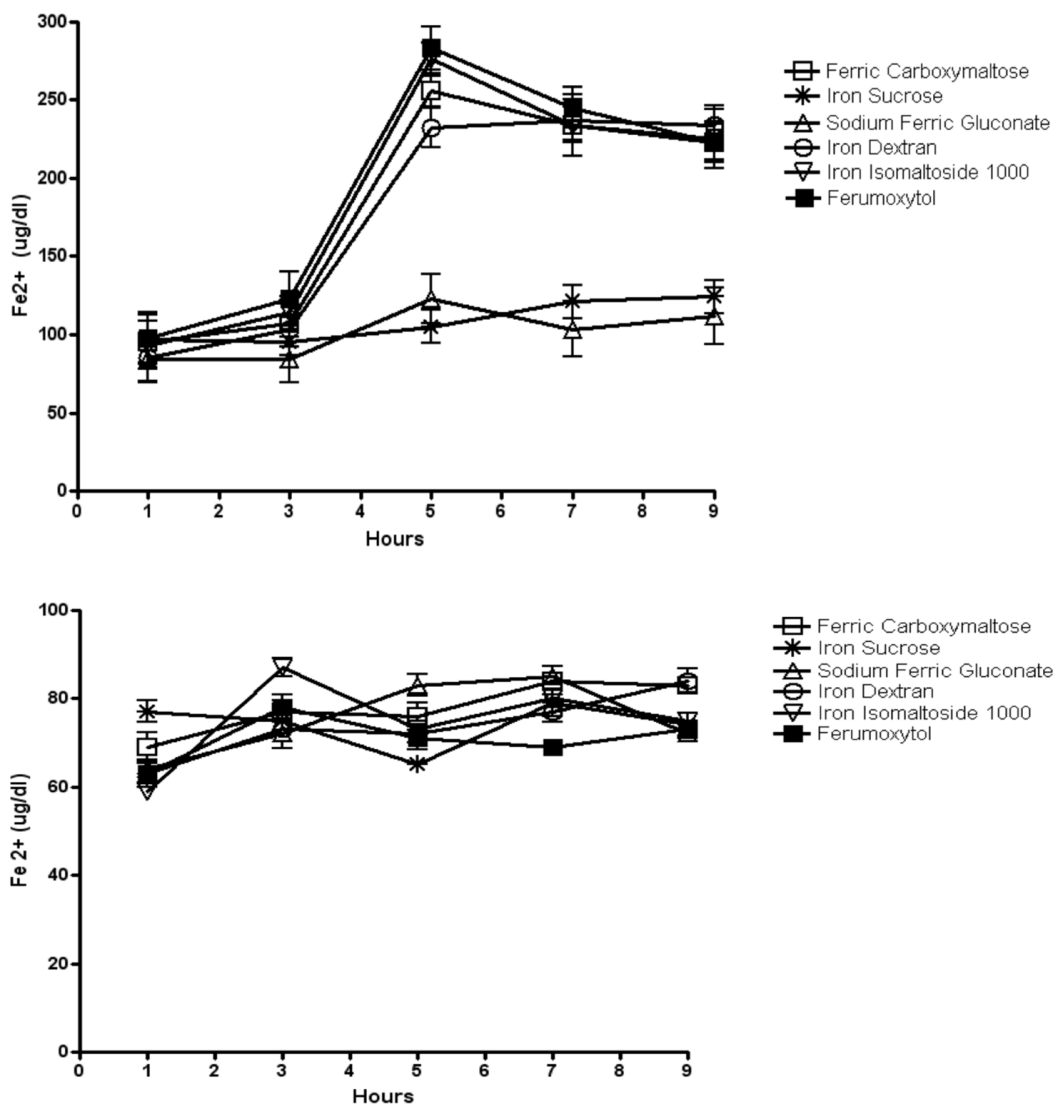
Ferrous iron content in media after exposure to the iron formulations with and without hepcidin treatment. Rat peritoneal macrophages were obtained by peritoneal lavage. The cells were exposed to 0.1 mg/ml of elemental iron for each of the iron formulations for 24 hours. The media was then replaced for 24 hours and then monitored for release of ferrous iron by removing 50uL aliquots from the media over 9 hours (top figure, A). In a separate experiment after 24 hours the media was replaced but included 500nM hepcidin. Following an additional 24 hours, an aliquot of the media was collected at the different time points shown on the graph (bottom figure, B). The increase in iron release seen previously (A) was blocked by hepcidin and the values for all of the iron compounds are less than that seen in A (B). It is noteworthy that the values on the y-axis of B are less than in A. Data represent the mean±SEM from three independent experiments.

The increase in ferritin expression by macrophages indicates that iron is being taken up and stored in the cells. However, when the iron is needed, macrophages release iron through the iron exporter ferroportin [14] or by secretion of ferritin [19]. Thus, we determined ferrous iron (soluble iron) content in media after treatment with the iron formulations. The results show that after 24 hours of exposure to the iron compounds the ferrous iron levels in the media are rising except for iron sucrose and sodium ferric gluconate. These relatively high levels of iron release for these compounds persist over the time period evaluated, whereas release of iron from macrophages exposed to iron sucrose and sodium ferric gluconate remains consistently low over the 9 hour time period evaluated (Fig 9A).

To determine if the release of iron from macrophages could be regulated, we exposed the cells to hepcidin. The presence of hepcidin in the media blocked the increase in ferrous iron in the media following exposure of the macrophages to the different iron formulations (Fig 9B).

**Fig 10:**
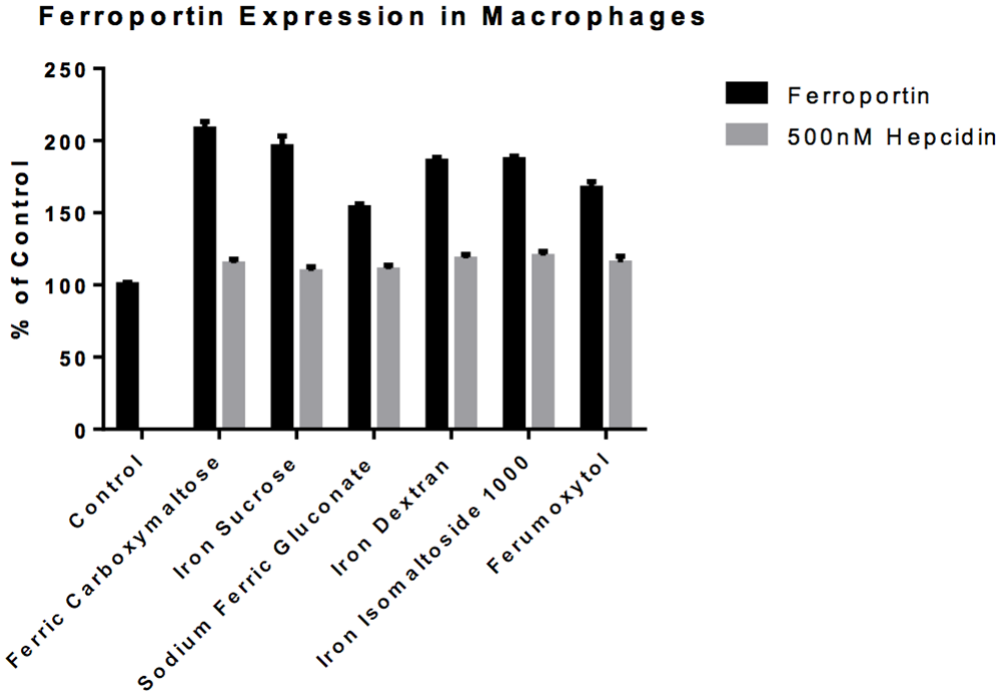
Ferroportin expression in Macrophages. Rat peritoneal macrophages were obtained as described in the methods. The cells were harvested after a 6-hour exposure to 0.1 mg/ml elemental iron from the different iron compounds in the presence or absence of 500nM hepcidin. The data show that exposure to all of the compounds is associated with an increase in ferroportin expression (band at 63 kDa not shown). The iron compound induced ferroportin expression was blunted by the presence of hepcidin. The expression levels were normalized to β-actin.

The response to hepcidin suggested that the iron export protein ferroportin was involved because hepcidin binds to ferroportin as the mechanism to block iron export from cells [20]. To address this possibility the first experiment was to demonstrate that ferroportin was expressed on the macrophages (Fig 10). Ferroportin expression was increased in the macrophages exposed to all of the iron compounds; the increase was least with sodium ferric gluconate. Following exposure of macrophages to hepcidin, the level of ferroportin expression in macrophages is decreased to control levels regardless of the iron formulation used (Fig 10), consistent with the iron release data (Fig 9B).

**Fig 11:**
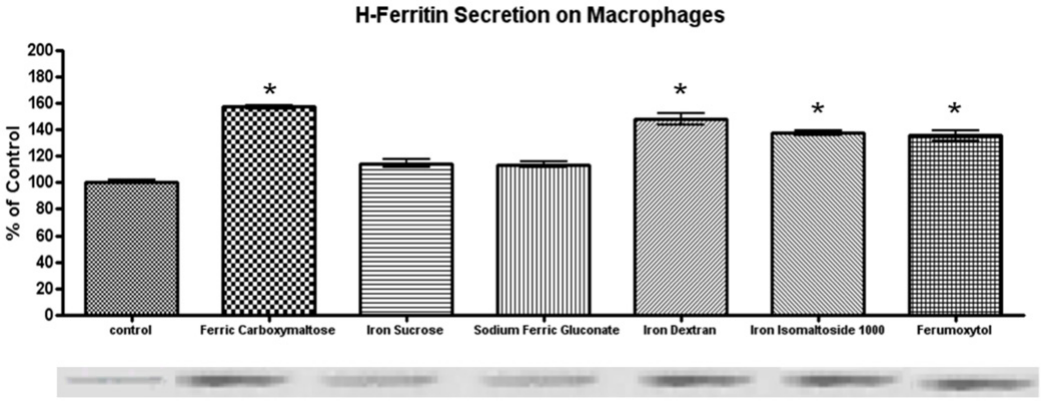
H-ferritin secretion by primary peritoneal macrophages after iron compound treatment for 24 hours: Rat peritoneal macrophages were obtained by peritoneal lavage. The cells were exposed to 0.1 mg/ml of elemental iron for each of the iron formulations for 24 hours. The media was then replaced for 24 hours and then an aliquot taken for H-ferritin analysis. A representative immunoblot (21 kDa band) is shown and the data for 3 experiments are presented in the graphs. The data show that exposure to ferric carboxymaltose, iron dextran, iron isomaltoside and ferumoxytol is associated with an increase in ferritin release by the macrophages compared to control (untreated) but there is no increase following iron sucrose or sodium ferric gluconate exposure (p<0.05). The expression levels were normalized to β-actin.

As mentioned, in addition to releasing iron via ferroportin, macrophages will release iron bound within ferritin that can be used to recycle iron [19] and possibly deliver iron via ferritin receptors if released as the H-subunit of ferritin [21]. Therefore, we determined the amount of H-ferritin in the media after 24 hours of exposure to the different iron compounds. All of the compounds except iron sucrose and sodium ferric gluconate were associated with increased levels of H-ferritin in the media (Fig 11).

### Effect of Iron Formulations on Cytokine Expression by Macrophages

**Fig 12:**
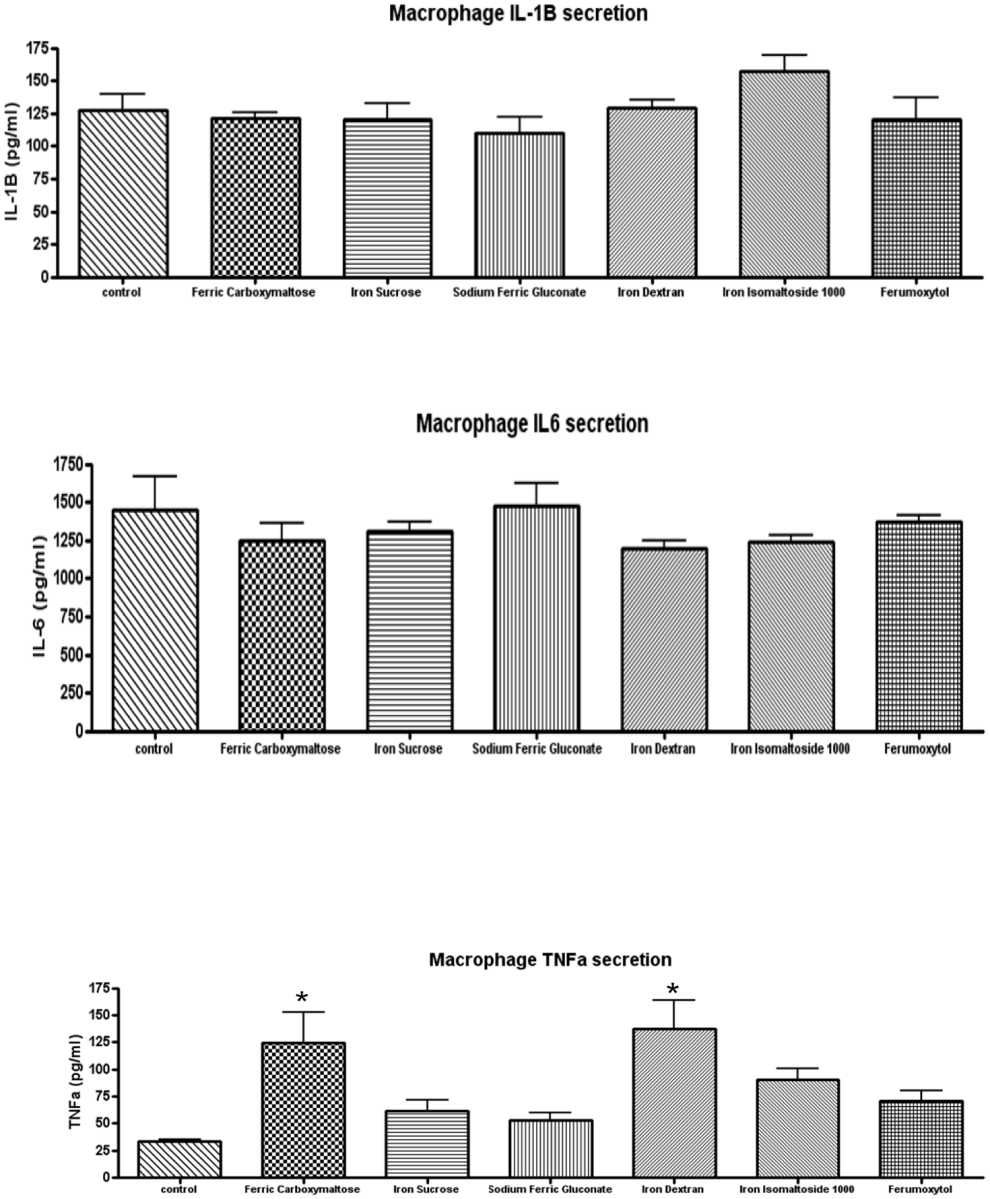
Cytokine secretion by primary peritoneal macrophages. Equal concentrations of 0.1 mg/ml elemental iron of the different iron compounds was added to the media. After 24 hours, the cells were rinsed and the media was changed to 2% fetal bovine serum in which the cells resided for another 24hour. The media was then collected and assayed for the different cytokines (A) Interleukin 1B, (B) Interleukin 6, (C) Tumor necrosis factor. All data shown are means ± SEM of three independent observations in separate cell culture wells. Differences were only seen with exposure to TNFα and only ferric carboxymaltose and iron dextran increases reached statistical significance *p<0.05 compared with control.

Macrophages express cytokines when activated and the proinflammatory cytokines can promote cytotoxicity and impact the immune response [14,22]. Therefore we determined the impact of the different iron formulations on the expression of interleukin 1B, interleukin 6 and tumor necrosis factor α (TNFα). The concentration of 0.1 mg/ml elemental iron was used for each of the experiments. Neither IL-1B nor IL-6 secretion was affected by any of the iron compounds (Fig 12A and 12B). TNFα was increased by ferric carboxymaltose and iron dextran (Fig 12C).

## Discussion

Intravenous iron formulations have increased in development and clinical application in recognition of the considerable medical need for an effective and in some cases, rapid treatment for iron deficiency anemia. Intravenous iron therapy provides a potentially rich and relatively rapid source of bioavailable iron, which improves serum hemoglobin and reduces the requirement for erythropoietin. There remains, however, considerable concern regarding the production and subsequent toxicity of bioactive iron [23]; i.e. reactive iron produced by the release of iron from the iron carbohydrate or nanoparticle complex that can induce oxidative damage via the Fenton Reaction. The mechanism by which the elemental iron in the various iron formulations becomes bioavailable is poorly understood. In this study we examined the cytotoxic potential of six pharmaceutical iron formulations on a human kidney cell line, rat renal cortical homogenates and rat peritoneal macrophages. The kidney was chosen for evaluation based on clinical concerns that it could be a target for damage due to its role in filtering serum and its relatively high lipid content. The peritoneal macrophages were chosen as representative of the reticuloendothelial system, which is the cell population most likely to initially attempt to store and subsequently release iron from the infusions. The rationale for this study is that the direct application of the iron compounds to cell culture media would provide a sensitive model, without confounding variables, for analysis of indices of oxidative stress and insights into the manner in which the iron compounds are handled by the macrophages.

The results reveal significant differences in the cytotoxicity profile and bioavailability profiles among the different iron compounds and the different cell models; although most compounds elicit some degree of oxidative stress. Moreover, the results also indicate that the release of iron from macrophages is significantly influenced by hepcidin. Hepcidin is elevated in inflammatory reactions and thus the release of iron from macrophages from an intravenous injection could be limited during inflammation in a manner similar to oral iron uptake from the gut. Some of the iron compounds, in low concentrations however, had proliferative effects, which is not unexpected given the iron requirements for cell growth.

### HK-2 Cells

Based on the LDH assay, a sensitive indicator of loss of cell membrane integrity, and the robust increase in lipid peroxidation at the lowest concentration, the HK-2 cells appear most sensitive to sodium ferric gluconate. The MTT assay for cell viability, however, did not show evidence that the impact of sodium ferric gluconate was lethal until the highest concentration of iron compounds. At the highest concentration, all of the compounds showed evidence of toxicity. Within the clinically relevant concentration range of 0.2–0.4 mg/ml for ferric carboxymaltose, LDH release was not detected. The MTT assay indicated cell viability was most sensitive to iron isomaltoside and ferumoxytol. The MTT assay suggested both iron dextran and iron isomaltoside had a mild but statistically significant proliferative effect at the lowest dose. Iron is needed for mitochondrial energy production and is a rate limiting step in production of ribonucleotide reductase needed for DNA synthesis [24]. Thus, the addition of iron in a bioavailable form could be expected to stimulate some cell proliferation. The dichotomous effect of these compounds being stimulatory at low concentrations and cytotoxic at higher concentrations would be consistent with a compound showing high bioavailability of the iron in the formulation. All of the compounds induced lipid peroxidation but based on the cell viability assay this damage was not part of a profile of lethality until the highest concentrations. Protein oxidative damage, as measured by oxidative modifications to protein, was only seen at the higher concentrations in two compounds indicating that the oxidative damage was directed at lipids, which are known to be more sensitive than proteins [25].

Previously, four of the compounds used in our study were examined in a similar cell culture model [26,27,28]. Our results are, in general, similar to published reports for iron sucrose and sodium ferric gluconate although iron sucrose was the more toxic of the two compounds in their study. A major difference from the published studies is that iron dextran was not toxic to HK-2 cells but was toxic in our study beginning at 0.2 mg/ml in the LDH assay. This difference could represent cell sensitivity to high and low molecular weight dextran. Our study used the higher molecular weight formulation whereas the other used the low molecular weight compound. The cell viability results for iron dextran were similar where loss of cell viability was only seen at the highest concentration [27]. Zagar et al (2010) examined ferumoxytol and found no loss of cell viability at 0.5 mg/ml but did find loss of viability at 1 mg/ml of compound. In our study, the HK-2 cells were more sensitive to ferumoxytol, which induced LDH release at beginning at 0.2 mg/ml and decreased cell viability at 0.4 mg/ml [28]. Similar to all of the iron compounds in our study, ferumoxytol exposure caused significant lipid peroxidation which indicated that there was ongoing oxidative stress, although HO-1 or MCP-1 did not increase in the HK-2 cells following ferumoxytol exposure [28]. This difference in oxidative stress response could represent differences in the type of cell stress pathways that were activated or could also be a technical difference in exposure times. In our study, the compound exposure was for longer (24hrs) than that reported in Zagar et al 2010 (18hrs) that could account for the effect at lower concentrations since the highest concentration did have an effect in the Zagar study.

### Renal Cortical Homogenates

In general, the renal cortical homogenates were more susceptible to the iron compounds than the HK-2 cells as measured by lipid peroxidation and oxidative modification to proteins. The different iron formulations were similar in effect except for iron dextran, which was significantly more potent than the other compounds at inducing lipid peroxidation at the shorter exposure time. The impact of iron dextran in our study was more potent that than previously reported [27] which again is likely due to the differences in low versus high molecular weight dextran. Iron sucrose did not induce lipid peroxidation at any concentration during the short exposure time but induced significant peroxidation at the lowest concentration after one hour of exposure. These results for sucrose are similar to those previously reported [27]. Ferumoxytol was the only compound not to induce lipid peroxidation after one hour of exposure. At the clinically relevant concentrations of ≤0.4mg only ferumoxytol and ferric carboxymaltose did not induce lipid peroxidation. These data suggest that the lipid peroxidation induced by these compounds was manageable by oxidative protective mechanisms. These data are also consistent with reports that ferumoxytol has no effect on LDH release following exposure to mouse proximal tubule cells. The impact of the iron compounds on protein oxidation was similar to that seen for lipids.

### Peritoneal Macrophages

The impact of the iron compounds on macrophages was investigated because these cells are likely to be the ones that first accumulate iron following intravenous injection [29]. The subsequent release of iron from the macrophages then becomes a key point in understanding the efficacy of the compounds. Moreover, the polarity of the macrophages will be influenced by the iron status of the cells [14, 30]. The analysis of iron compound management by macrophages revealed significant differences among the different iron formulations. Iron isomaltoside and ferumoxytol appeared to have mild stimulatory effects on the macrophages at low doses similar to their effect on the HK-2 cells. Sodium ferric gluconate induced LDH release at the lowest concentration although it did not decrease cell viability. The uptake of sodium ferric gluconate by macrophages was relatively poor which was consistent with the poor release of iron. Iron sucrose was less toxic to macrophages than sodium ferric gluconate but had a similar profile of poor uptake. Iron dextran and iron isomaltoside also induced LDH release at the clinically relevant concentrations and all of the compounds except ferric carboxymaltose eventually induced LDH release. However, only iron sucrose was associated with decreased cell viability. These findings may translate to the in vivo findings that both iron sucrose and sodium ferric gluconate induce significant levels of lipid peroxidation in plasma and renal cortex following intravenous injection in rats [26].

Macrophages are key players in the immune response through multiple pathways including the release of proinflammatory cytokines. Iron accumulation in macrophages may underlie their activation to the M1 proinflammatory phenotype [14,30]. Therefore we investigated the impact of the different iron compounds on the release of inflammatory cytokines by macrophages. Of the 3 cytokines examined, only secretion of TNFα was increased and this increase only occurred in response to exposure to ferric carboxymaltose and iron dextran. An increase in TNFα was observed following intravenous injections of iron sucrose, sodium ferric gluconate and iron dextran in a rat model [31] but the effect was studied in conjunction with a LPS injection to activate the macrophages; whereas our study was focused on the effect of the iron compounds alone. In a study on rats receiving intravenous iron sucrose, the peritoneal macrophages were removed and found to release increased levels of tumor necrosis factor α following exposure to endotoxin in vitro [32]. Our data show a TNFα response can be induced by two of the compounds independent of LPS activation.

As mentioned, macrophages are expected to release the iron accumulated from the different iron compounds in a bioavailable form. There are two mechanisms of iron secretion by macrophages, secretion as ferritin and release of ferrous iron through the iron export protein ferroportin [14,19,30]. We have previously shown that the H-ferritin subunit is taken up by all organs, including the brain, at a higher rate than L-ferritin [21]. Thus, we focused our analysis on H-ferritin and found that all of the iron compounds except iron sucrose and sodium ferric gluconate were associated with an increase in release of H-ferritin. Although the Calcein assay indicated that iron sucrose and sodium ferric gluconate exposure was associated with increased labile iron in the macrophages there was less stimulation of ferritin. Thus, the decrease in H-ferritin release is consistent with the relatively less impact on ferritin synthesis for these two compounds. The combined effect of decreased ferritin stimulation and decreased iron release by macrophages suggests that iron sucrose and sodium ferric gluconate may reflect differences in the construction of the iron core that make the reduction from Fe+3 to Fe+2 kinetically more difficult. This interpretation requires consideration of two points. First, calcein may bind Fe+3 [33] so the increase in LIP could occur without reduction to Fe+2 (Fig 7). Secondly, an increase in Fe+3 in the cell, as suggested by the LIP analysis could be expected to induce oxidative stress, but Fe+3 would not engage in the Fenton reaction [34]. Thus, even though there is an increase in the LIP without an increase in ferritin, the lack of an increase in oxidative stress in the macrophages exposed to iron sucrose and sodium ferric gluconate is not inconsistent with increased Fe+3 in the LIP. The data therefore suggests that iron from iron sucrose and sodium ferric gluconate is less biologically available.

Release of ferrous iron into the media was similar to the profile for H-ferritin release. Ferrous iron release is via the iron export protein ferroportin [11]. We demonstrated that ferroportin was present on the macrophages and ferroportin expression was increased following exposure to all of the compounds. The iron release via ferroportin can be blocked by hepcidin, an iron regulatory hormone released by liver cells. Moreover, the presence of hepcidin will decrease expression of ferroportin. This observation is noteworthy because a leading clinical argument for choosing an intravenous approach over oral supplementation during inflammation is that the iron absorption from the gut will be limited because hepcidin is elevated in the plasma during inflammation. The notion is that elevated hepcidin will bind to ferroportin on the enterocytes and limit iron uptake into the body [35,36]. Our data suggest a portion of the iron could be held within the macrophages via the same mechanism.

Our studies on iron uptake in macrophages differs from that reported for HEPG2 cells [37] that take up iron from iron sucrose and iron gluconate more readily than low molecular weight iron dextran. But, the comparison between a cell line and primary macrophages is difficult, and the iron dextran was different from that used in the current study. Two formulations of iron dextran and a number of iron hydroxyethyl starch compounds did not increase ferritin in a human macrophage cell line [37] but the impact of the iron formulations on cell proliferation in the cell line models was not determined and could account for the decreased ferritin content in the cells which was normalized to total protein. Release of iron following exposure to different iron dextran formulations and the iron hydroxyethyl starch compounds was reported in HEP2G and THP-1 macrophage cell lines similar to our findings but there was no difference in the amount of iron released for these compounds [38].

Although extrapolation to clinical use from a cell culture model requires considerable caution, the cell culture model provides a means of assessment of direct exposure to cells, representing the most challenging situation at the cellular level. The results of this study have shown that the compounds can differ in their cellular uptake, induction of markers of oxidative stress and release of inflammatory cytokines. Some of the compounds can induce cell proliferation at low doses. The data clearly demonstrates that iron released from macrophages is possible in two modalities following uptake of iron from the various compounds. The release of iron via ferroportin is regulated by hepcidin and may work to diminish some of the effect of the intravenous iron.
